# Two new *Eresus* species (Araneae, Eresidae) from Xinjiang, China

**DOI:** 10.3897/BDJ.10.e94853

**Published:** 2022-12-06

**Authors:** Yejie Lin, Shuqiang Li, Xin Zhao, Zhanqi Chen, Haifeng Chen

**Affiliations:** 1 Hebei Key Laboratory of Animal Diversity, College of Life Science, Langfang Normal University, Langfang, China Hebei Key Laboratory of Animal Diversity, College of Life Science, Langfang Normal University Langfang China; 2 Institute of Zoology, Chinese Academy of Sciences, Beijing, China Institute of Zoology, Chinese Academy of Sciences Beijing China; 3 CAS Key Laboratory of Tropical Forest Ecology, Xishuangbanna Tropical Botanical Garden, Chinese Academy of Sciences, Mengla, China CAS Key Laboratory of Tropical Forest Ecology, Xishuangbanna Tropical Botanical Garden, Chinese Academy of Sciences Mengla China

**Keywords:** Asia, diagnosis, DNA barcodes, taxonomy, type

## Abstract

**Background:**

Eresidae C. L. Koch, 1845 contains nine genera and 102 species, of which 24 species belong to *Eresus* Walckenaer, 1805. Four species of the family are known from China: *E.granosus* Simon, 1895 (Beijing), *E.kollari* Rossi, 1846 (Hebei), *E.lishizheni* Lin, Marusik & Li, 2021 (Xinjiang) and *Stegodyphustibialis* (O. Pickard-Cambridge, 1869) (Yunnan).

**New information:**

Two new species of *Eresus* are described from Xinjiang, China: *Eresusda* Lin & Li sp. n. and *E.yukuni* Lin & Li sp. n. Photos and morphological descriptions of new species are given.

## Introduction

The spider family Eresidae C. L. Koch, 1845, commonly known as velvet spiders, is almost entirely limited to the Old World, with the exception of one species known from Brazil (World Spider Catalog 2022). *Eresus* Walckenaer 1805 are distributed in the Palaearctic and live in dry areas with short vegetation and well-drained soil. *Eresus* spiders are attracting public attention due to their striking colours. They are listed as a protected species, such as *E.sandaliatus* (Martini & Goeze, 1778) in the United Kingdom and *Eresus* spp. in Poland (Milano et al. 2021).

Chinese spider taxonomists have published a large number of papers in the 21^st^ century, but due to the rich biodiversity of the Chinese territory, there are still many unknown species (Li et al. 2021, Lin et al. 2021, Li 2020, Yao et al. 2021, Zhao et al. 2022). In the current paper, we describe two new *Eresus* species from Xinjiang, China: *E.da* sp. n. and *E.yukuni* sp. n.

## Materials and methods

Morphological and ecological data

Type specimens were preserved in 80% ethanol. The spermathecae were cleared in trypsin enzyme solution to dissolve non-chitinous tissues. Specimens were examined under a LEICA M205C stereomicroscope. Photomicrographs were taken with an Olympus C7070 zoom digital camera (7.1 megapixels). Laboratory habitus photographs were taken with a Sony A7RIV digital camera, equipped with a Sony FE 90 mm Goss lens. Photos were stacked with Helicon Focus® (Version 7.6.1) or Zerene Stacker® (Version 1.04) and processed in Adobe Photoshop CC2019^®^.

All measurements are in millimetres (mm) and were obtained with an Olympus SZX16 stereomicroscope with a Zongyuan CCD industrial camera. Measurements of body lengths do not include the chelicerae. Eye sizes are measured as the maximum diameter from either the dorsal or frontal view. Leg measurements are given as follows: total length (femur, patella+tibia, metatarsus, tarsus). Abbreviations: ALE, anterior lateral eyes; AME, anterior median eyes; BH, basal haematodocha; C, conductor; CD, copulatory duct; E, embolus; F, fissure; FD, fertilisation duct; G, groove; L, lamella; MH, median haematodocha; PLE, posterior lateral eyes; PME, posterior median eyes; S, spermatheca; SD, sperm duct; Sh, shoulder; ST, subtegulum; T, tegulum; TT, terminal tooth. References to figures in cited papers are in lowercase (fig. or figs.) and figures in this paper are noted with an initial capital (Fig. or Figs.). The terminology used in the text and figures follows Řezáč, Pekár and Johannesen (2008) and Krejčí, Řezáč and Michalik (2015) (Řezáč et al. 2008, Krejčí et al. 2015).

Type materials are deposited in the Institute of Zoology, Chinese Academy of Sciences in Beijing (**IZCAS**).

Sequences of 20 Eresidae specimens were downloaded from the National Center for Biotechnology Information (NCBI) and a further two are based on both holotypes in this study. Whole genomic DNA was extracted from 2–4 legs using a TIANamp Genomic DNA kit (TIANGEN Inc., Beijing, China) following the manufacturer’s protocol. One gene fragment was amplified in 20-μl reactions: COI (~ 640 bp). Primers and PCR conditions follow Folmer et al. (1994) and Hedin et al. (2001). All sequences were analysed using BLAST and are deposited in GenBank. Sequence alignment was performed in MAFFT V.7.313. The K2P distance of Intra-specific and inter-specific nucleotide divergences were calculated in MEGA.7.0. (Folmer et al. 1994, Hedin and Madison 2001, Kumar et al. 2016).

## Taxon treatments

### Eresus da

Lin & Li sp. n.

3A3D7EFE-07DE-52FB-A0E5-92ED4823B433

A52C7C88-A951-4E80-8B28-751DDF692327

#### Materials

- **Type status:** Holotype. **Occurrence:** recordedBy: Xin Zhao; individualID: IZCAS-Ar43547; sex: female; occurrenceID: 9B247645-5769-574E-9924-00D870022C35; **Taxon:** taxonID: urn:lsid:zoobank.org:act:A52C7C88-A951-4E80-8B28-751DDF692327; scientificName: *Eresusda* Lin & Li, sp. n.; kingdom: Animalia; phylum: Arthropoda; class: Arachnida; order: Araneae; family: Eresidae; genus: Eresus; **Location:** country: China; stateProvince: Xinjiang Uygur Autonomous Region; county: Changji Hui Autonomous Prefecture; municipality: Fukang City; locality: Wutonggou National Desert Park; verbatimElevation: 388 m; decimalLatitude: 44.3920; decimalLongitude: 87.8707; **Identification:** identifiedBy: Yejie Lin; **Event:** year: 2022; month: 7; day: 25

#### Description

Female (Holotype, IZCAS-Ar43547): Habitus as in Fig. 2A and B. Carapace 10.88 long, 8.62 wide, 8.92 high. Carapace nearly equally wide at pars cephalica and pars thoracica. Carapace red-brown with white setae; pars cephalica elevated. Eye sizes and interdistances: AME 0.21, ALE 0.26, PME 0.51, PLE 0.33, AME–AME 0.37, AME–ALE 3.56, PME–PME 0.65, PME–PLE 2.52, AME–PME 0.11. Pars cephalica with a pointed posterior margin dorsally, almost as long as wide. Chelicerae covered with narrowed white and black setae. Legs with a white ring of setae at joints. Legs with ventral macrosetae on Ta, Mt and Ti I–IV. Leg measurements: I: 17.83 (5.17+5.90+3.94+2.82); II: 16.46 (5.54+5.89+2.94+2.09); III: 14.77 (4.80+5.68+2.63+1.66); IV: 19.53 (6.33+7.29+3.74+2.17). Abdomen dark brown, covered with black setae, with white spots, sigilla conspicuous, surrounded by white setae.

Epigyne (Fig. 3) with sclerotised margins, longer than high. Middle plant beyond posterior edge of epigyne margin. Fissure bow-shaped. Copulatory duct translucent, with spermathecal epithelium on anterior edge. Spermathecae distinctly lobed, reaching further laterally than copulatory ducts.

Male. Unknown.

#### Diagnosis

Females of *Eresusda* sp. n. are similar to those of *E.kollari* by a relatively gentle slope of the ocular area (Fig. 2C and D; Řezáč et al. 2008, fig. 4D; Miller et al. 2012, fig. 2F) and with the epigynal area longer than high (Fig. 3A; Řezáč et al. 2008, figs. 2A–J). This species can be distinguished from *E.kollari* by the abdomen with a large number of white spots (Fig. 2D) (vs. abdomen uniformly black in *E.kollari*), the anterior edge of copulatory duct within the anterior edge of epigyne (vs. beyond the anterior edge of epigyne in *E.kollari*) and the ventral of fissure curved (vs. almost straight in *E.kollari*) (Fig. 4).

#### Etymology

The species name is a noun in apposition derived from the Chinese pinyin “dà” (giant) and refers to the large size of this new species.

#### Distribution

Known only from the type locality (Xinjiang, China).

#### Ecology

**Figure 1.**
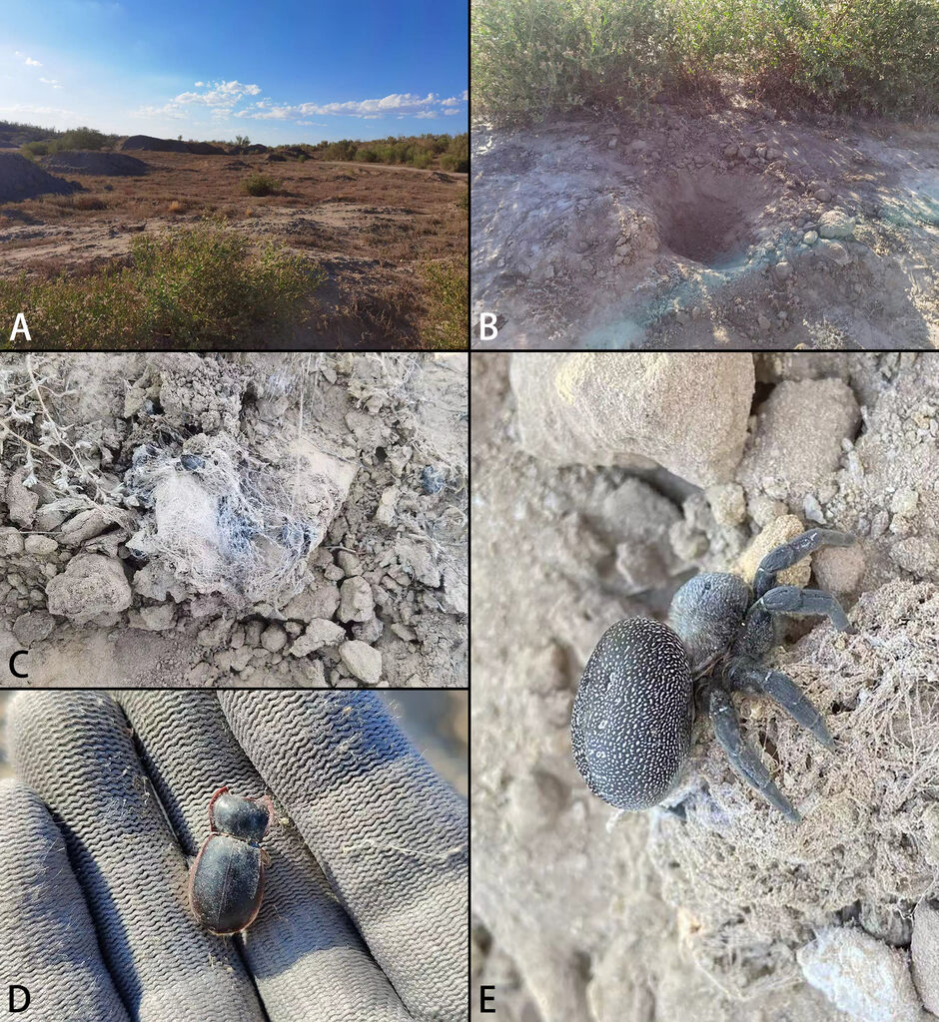
*Eresusda* sp. n. holotype female. **A** habitat; **B** microhabitat; **C** tunnel wrapped with beetle skeleton; **D** beetle skeleton (Pimeliinae sp.); **E** spider with nest.

The spider was found behind a clump of desert plants (Fig. 1A). The spider habitat is under the ground, with a silk tunnel (around 20 cm in length) connecting its opening and underground nest (Fig. 1A and B). This underground living strategy may be an adaptive strategy for escaping from the sunlight heating and extremely high temperature of the ground surface. As the silken tunnel was wrapped with dozens of empty beetle exoskeletons (Fig. 1C and D), we assume that the desert-living beetles are the main diet of the spider, which has a thick fang to pierce the beetles while feeding. In addition, the small size of the spider’s eyes suggest that the spider may adopt a sit-and-wait hunting strategy and that it does not rely on visual signals for hunting.

### Eresus yukuni

Lin & Li sp. n.

E57D02A7-39B0-538C-889A-E33AA7DDD18D

95651052-1B7C-47FE-8E6E-1D6D20E51B0A

#### Materials

- **Type status:** Holotype. **Occurrence:** recordedBy: Kun Yu; individualID: IZCAS-Ar43546; occurrenceID: F2E880E4-6CA6-5E5A-929E-63D1B3CE4703; **Taxon:** taxonID: urn:lsid:zoobank.org:act:95651052-1B7C-47FE-8E6E-1D6D20E51B0A; scientificName: *Eresusyukuni* Lin & Li, sp. n.; kingdom: Animalia; phylum: Arthropoda; class: Arachnida; order: Araneae; family: Eresidae; genus: Eresus; **Location:** country: China; stateProvince: Xinjiang Uygur Autonomous Region; municipality: Urumqi City; locality: Saybag District, near Shihuoshan Tunnel; decimalLatitude: 43.7947; decimalLongitude: 87.4783; **Identification:** identifiedBy: Kun Yu; **Event:** year: 2019; month: 6; day: 24

#### Description

**Figure 2.**
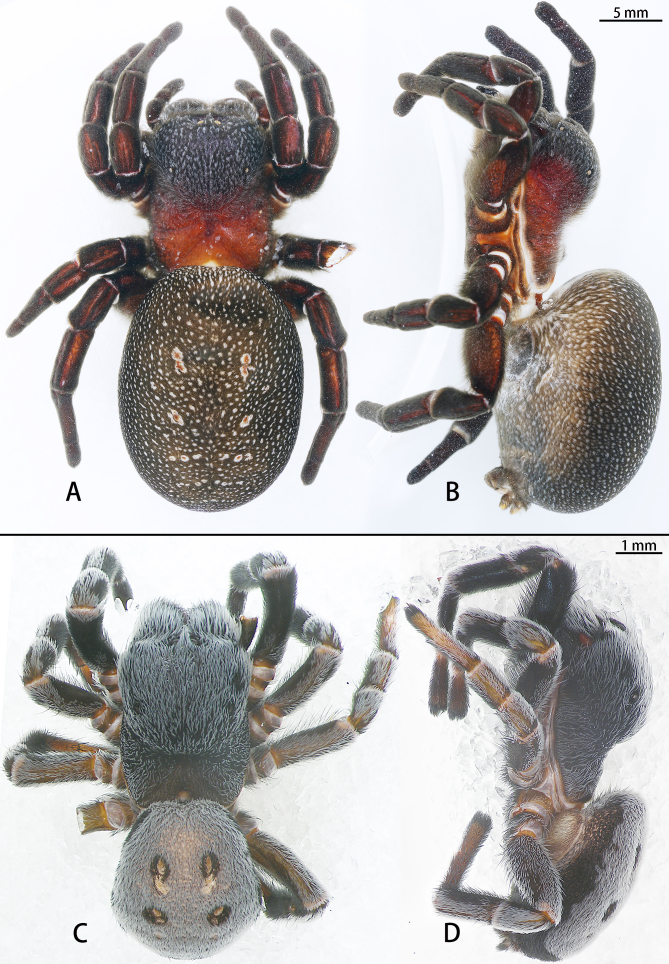
*Eresusda* sp. n. holotype female (**A**, **B**) and *E.yukuni* sp. n. holotype male (**C**, **D**), habitus. **A, C** dorsal view; **B, D** lateral view.

**Figure 3.**
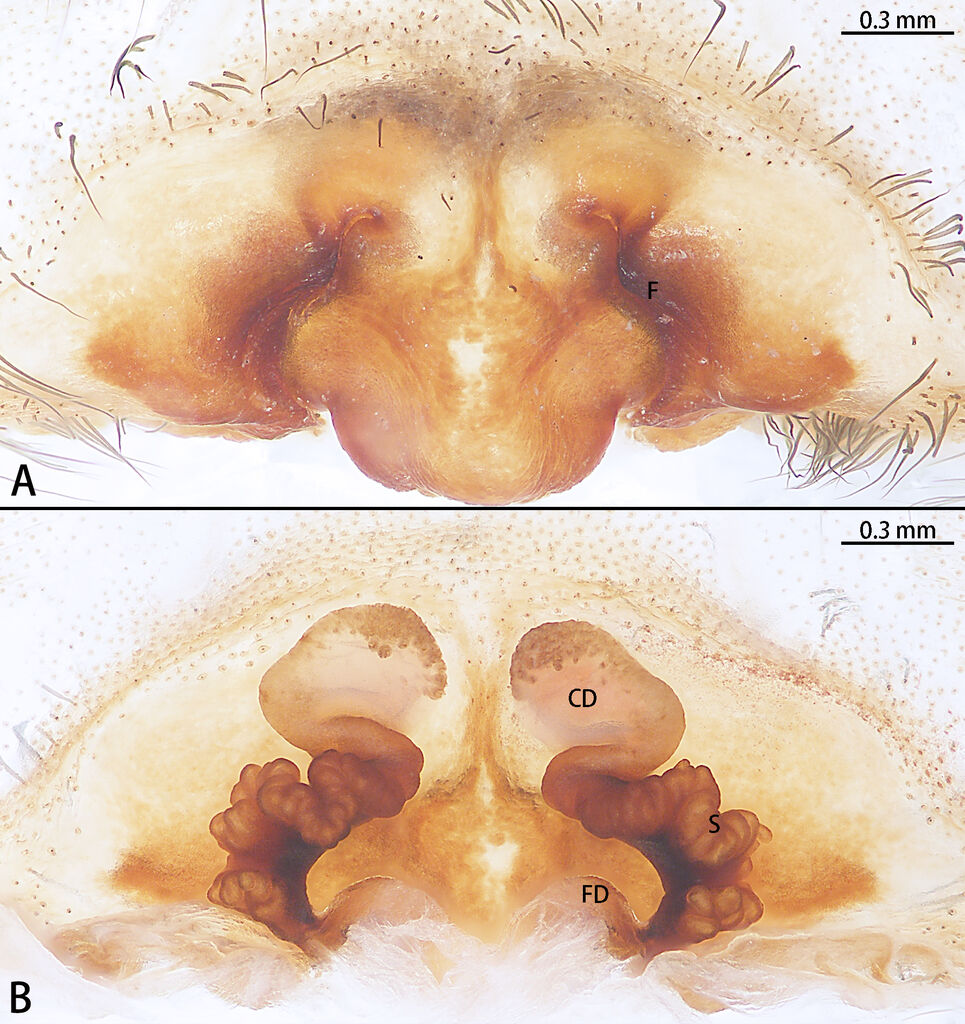
*Eresusda* sp. n. holotype female **A** epigyne, ventral view; **B** vulva, dorsal view.

**Figure 4.**
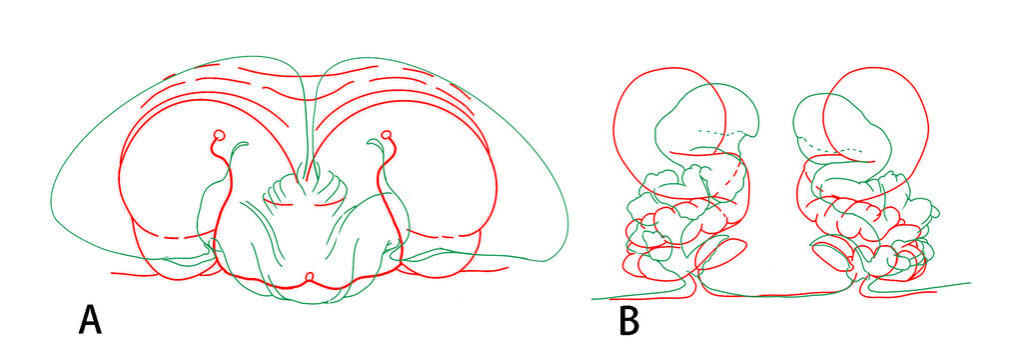
*Eresus* spp., outlines of copulatory organs (green line, *Eresusda* sp. n.; red line, *E.kollari*, changed after Řezáč et al. (2008). **A** epigyne, ventral view; **B** vulva, dorsal view.

Male (Holotype, IZCAS-Ar43546): Habitus as in Figs. 2C and D. Total length: 6.48, carapace 3.35 long, 2.44 wide, 1.76 high. Abdomen 3.20 long, 2.88 wide. Carapace black with white setae; pars cephalica elevated. Eye sizes and interdistances: AME 0.09, ALE 0.13, PME 0.18, PLE 0.08, AME–AME 0.12, AME–ALE 0.78, PME–PME 0.19, PME–PLE 1.03, AME–PME 0.04, ALE–PLE 1.88. Pars cephalica with a pointed posterior margin dorsally, almost as long as wide (2.54 long, 2.83 wide). Chelicerae covered with black and few white setae. Leg I with a white ring of setae at joints, black; leg II–IV with white setae dorsally. Leg measurements: Leg I 6.54 (1.95+2.15+1.32+1.12); leg II 5.11 (1.51+1.67+1.00+0.93); leg III 4.55 (1.47+1.50+0.81+0.77); leg IV 6.12 (1.93+2.22+1.14+0.83). Abdomen dark brown, with four black spots, covered with dense white setae dorsally; ventrum with black setae, sigilla conspicuous, surrounded by black setae.

**Figure 5.**
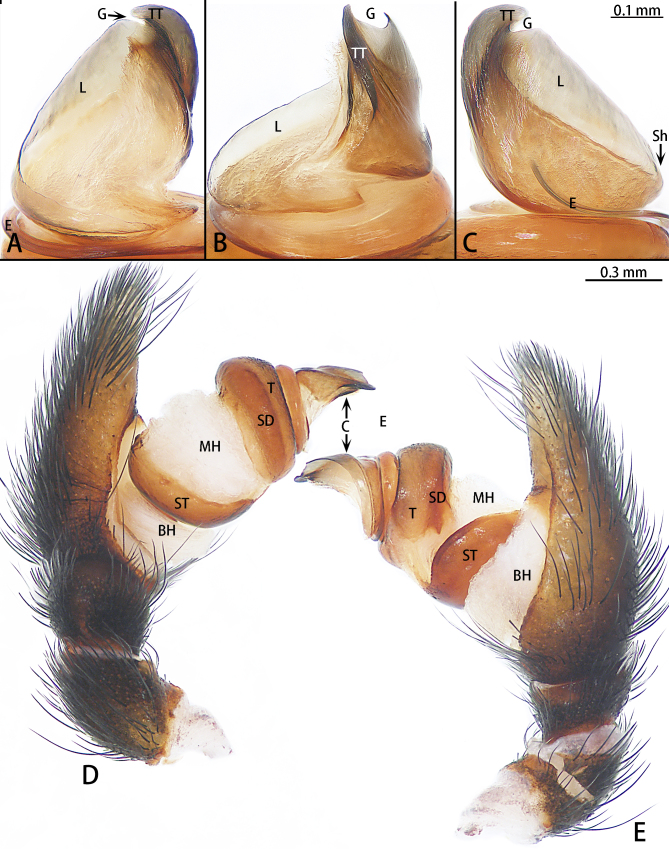
*Eresusyukuni* sp. n., holotype, right palp (Horizontal flip). **A** conductor, prolateral view; **B** same, ventral view; **C** same, retrolateral view; **D** palp, prolateral view; **E** same, retrolateral view.

Palp (Fig. 5). Tegulum round. Conductor height is greater than width, with a slight shoulder and curved terminal tooth; the height of the lamella is almost twice the length of terminal tooth; groove deep and narrow.

Female. Unknown.

#### Diagnosis

Male of *Eresusyukuni* sp. n. is similar to the *E.lavrosiae* Mcheidze, 1997 by having black carapace covered with short black and white setae that is almost as wide at the pars cephalica as it is at the pars thoracica (Fig. 2A and B; Zamani et al. 2020, fig. 15). However, this species can be distinguished from *E.lavrosiae* by the abdomen with dense white setae dorsally (Fig. 2A) [vs. with a frontally abrupt white circle in *E.lavrosiae* (Zamani et al. 2020, fig. 15)]. The palps are similar to those of *E.lavrosiae* by the strongly curved terminal tooth and the conspicuously deep lamellar groove, but can be distinguished by the shoulder near the base of the embolus in retrolateral view (Fig. 5B) (vs. shoulder far from the base of the embolus in *E.lavrosiae* (Zamani et al. 2020, fig. 19) and the conductor higher than wide in ventral view (Fig. 5C) [vs. wider than high in *E.lavrosiae* (Zamani et al. 2020, fig. 18)].

#### Etymology

The species is named after Mr. Kun Yu, who collected the holotype; noun (name) in the genitive case.

#### Distribution

Known only from the type locality (Xinjiang, China).

## Analysis

In this paper, we studied the COI sequences for 22 *Eresus* specimens (Table 1). Calculation of the K2P distance using MEGA.7.0, revealed that the new species had a significant genetic gap with other known species (Table 2). The interspecific genetic distance ranged from 4.5% to 14.5%, as shown in Table 2. Based on the 632 bp-aligned sequences, the COI uncorrected K2P-distance between *Eresusda* sp. n. and *E.kollari* is 7.3%, between *E.da* sp. n. and *E.moravicus* Řezáč, 2008 is 6.6% and between *Eresusda* sp. n. and *E.yukuni* sp. n. is 8.7%. The result exceeded the maximum value of intraspecific genetic distance for Eresidae.

## Supplementary Material

XML Treatment for Eresus da

XML Treatment for Eresus yukuni

## Figures and Tables

**Table 1.** List of voucher information and GenBank accession numbers of 22 *Eresus* specimens.

**Taxon**	**Location**	**GenBank Accession Number**
*E.moravicus*	NA	MH673855.1
*E.kollari*	NA	KX537083.1
*E.walckenaeri*	NA	FJ948999.1
E.cf.kollari	NA	FJ948998.1
*E.* sp. 1306	NA	FJ948997.1
*E.* sp. 2	NA	OL352216.1
*E.* sp. H	NA	OL352217.1
*E.hermani*	NA	OL352220.1
*E.* sp. 1	NA	OL352221.1
*E.kollari*	NA	OL352223.1
*E.lavrosiae*	NA	OL352224.1
*E.hermani*	NA	OL352225.1
*E.kollari*	NA	OL352226.1
*E.* sp. D	NA	OL352228.1
*E.walckenaeri*	NA	OL352229.1
*E.* sp. F	NA	OL352230.1
*E.sandaliatus*	NA	OL352231.1
*E.kollari*	NA	OL352232.1
*E.crassitibialis*	NA	OL352233.1
E.cf.kollari	NA	OL352235.1
*E.da* sp. n.	China, Xinjiang, Changji	OP376824
*E.yukuni* sp. n.	China, Xinjiang, Urumqi	OP434393

**Table 2.** Estimates of evolutionary divergence between *Eresus* spp.

	MH673855.1	KX537083.1	FJ948999.1	FJ948998.1	FJ948997.1	OL352216.1	OL352217.1	OL352220.1	OL352221.1	OL352223.1	OL352224.1	OL352225.1	OL352226.1	OL352228.1	OL352229.1	OL352230.1	OL352231.1	OL352232.1	OL352233.1	OL352235.1	*E.da* **sp. n.**	*E.yukuni* **sp. n.**
MH673855.1																						
KX537083.1	0.050																					
FJ948999.1	0.100	0.102																				
FJ948998.1	0.090	0.093	0.101																			
FJ948997.1	0.114	0.125	0.111	0.118																		
OL352216.1	0.043	0.055	0.120	0.087	0.116																	
OL352217.1	0.041	0.033	0.096	0.095	0.114	0.046																
OL352220.1	0.051	0.050	0.114	0.079	0.105	0.023	0.048															
OL352221.1	0.089	0.084	0.126	0.102	0.134	0.085	0.089	0.081														
OL352223.1	0.045	0.006	0.105	0.093	0.125	0.055	0.035	0.050	0.084													
OL352224.1	0.069	0.061	0.103	0.095	0.122	0.068	0.065	0.072	0.084	0.069												
OL352225.1	0.053	0.053	0.115	0.084	0.113	0.023	0.048	0.006	0.082	0.053	0.072											
OL352226.1	0.050	0.008	0.097	0.082	0.127	0.053	0.035	0.048	0.078	0.011	0.067	0.052										
OL352228.1	0.078	0.077	0.117	0.063	0.133	0.074	0.082	0.072	0.091	0.073	0.083	0.076	0.071									
OL352229.1	0.010	0.111	0.032	0.101	0.113	0.108	0.105	0.105	0.131	0.106	0.108	0.106	0.109	0.118								
OL352230.1	0.057	0.057	0.110	0.010	0.130	0.020	0.055	0.025	0.094	0.057	0.077	0.025	0.059	0.087	0.099							
OL352231.1	0.055	0.055	0.116	0.087	0.117	0.027	0.053	0.019	0.089	0.055	0.083	0.019	0.053	0.076	0.101	0.028						
OL352232.1	0.045	0.005	0.102	0.090	0.122	0.055	0.035	0.050	0.084	0.002	0.069	0.053	0.001	0.073	0.106	0.057	0.055					
OL352233.1	0.133	0.122	0.130	0.111	0.145	0.129	0.120	0.126	0.134	0.120	0.134	0.127	0.116	0.133	0.124	0.137	0.131	0.118				
OL352235.1	0.072	0.067	0.103	0.092	0.108	0.077	0.070	0.077	0.085	0.069	0.062	0.074	0.066	0.080	0.113	0.079	0.077	0.067	0.113			
*E.da* sp. n.	0.075	0.081	0.108	0.077	0.131	0.082	0.083	0.082	0.096	0.083	0.086	0.082	0.076	0.088	0.103	0.096	0.086	0.081	0.118	0.075		
*E.yukuni* sp. n.	0.083	0.091	0.100	0.095	0.116	0.096	0.085	0.090	0.106	0.091	0.070	0.091	0.091	0.086	0.108	0.097	0.095	0.091	0.131	0.061	0.087	
